# The amphioxus *(Branchiostoma floridae) *genome contains a highly diversified set of G protein-coupled receptors

**DOI:** 10.1186/1471-2148-8-9

**Published:** 2008-01-16

**Authors:** Karl JV Nordström, Robert Fredriksson, Helgi B Schiöth

**Affiliations:** 1 Department of Neuroscience, Functional Pharmacology, Uppsala University, BMC, Box 593, 751 24, Uppsala, Sweden

## Abstract

**Background:**

G protein-coupled receptors (GPCRs) are one of the largest families of genes in mammals. *Branchiostoma floridae *(amphioxus) is one of the species most closely related species to vertebrates.

**Results:**

Mining and phylogenetic analysis of the amphioxus genome showed the presence of at least 664 distinct GPCRs distributed among all the main families of GPCRs; *Glutamate (18)*, *Rhodopsin (570)*, *Adhesion (37)*, *Frizzled (6) *and *Secretin (16)*. Surprisingly, the *Adhesion *GPCR repertoire in amphioxus includes receptors with many new domains not previously observed in this family. We found many *Rhodopsin *GPCRs from all main groups including many amine and peptide binding receptors and several previously uncharacterized expansions were also identified. This genome has however no genes coding for bitter taste receptors (TAS2), the sweet and umami (TAS1), pheromone (VR1 or VR2) or mammalian olfactory receptors.

**Conclusion:**

The amphioxus genome is remarkably rich in various GPCR subtypes while the main GPCR groups known to sense exogenous substances (such as Taste 2, mammalian olfactory, nematode chemosensory, gustatory, vomeronasal and odorant receptors) in other bilateral species are absent.

## Background

The superfamily of G protein-coupled receptors (GPCRs) is one of the largest families of proteins in mammals [1]. GPCRs participate in a tremendous diversity of physiological functions by playing a key role in the mediation of extracellular signals into cells while approximately 50% of all marketed drugs act on GPCRs [2]. The superfamily can be divided according to the GRAFS system into five main families; *Glutamate*, *Rhodopsin*, *Adhesion*, *Frizzled *and *Secretin *[3]. These main families arose prior the split of nematodes from the chordate lineage. The *Rhodopsin *family has been very successful in evolutionary terms, representing about 60% of the GPCRs repertoire of eight bilateria species. However, the *Rhodopsin *family seems not to be present in *Dictyostelium discoideum *[4-6]. The *Rhodopsin *family can be divided in four main groups (α-, β- γ- and δ) with 13 main branches and previous studies have shown that members within each of the four main groups seem to be found in most bilaterial species, while the representation of each of the main branches is highly variable [4,6-8].

Amphioxus (*Branchiostoma floridae*) is a small cephalochordate that spends much of its time buried in the sand. It is one of the closest now living relatives to vertebrates. Amphioxus shares several features with vertebrates like a dorsal, hollow nerve cord, notochord, segmental muscles and pharyngeal gill slits. On the other hand, they are missing the pronounced head region of vertebrates as well as not having neural crest cells functioning similar to those in vertebrates, paraxial skeletal tissue and some visceral organs [9]. Lately it has been argued on the basis of molecular phylogenetic data that tunicates, such as *Ciona intestinalis*, could be more closely related to vertebrates than cephalochordates are [10]. GPCRs have been mined from the *C. intestinalis *genome while very little has been known about GPCRs in the most basal Cephalochordates such as amphioxus. However, this genome has just recently been sequenced and the first genome paper will soon be published. Version 1.0 is released in the form of scaffolds on the Joint Genome Institute homepage [11]. It has been predicted that this genome will be very important for the understanding of the evolution of many vertebrate gene families.

We have performed a detailed mining and phylogenetic analysis of the gene repertoire of GPCRs in amphioxus. We found that this genome has a highly diversified set of genes coding for GPCRs which provides insight into evolutionary aspects of these.

## Results & Discussion

**Figure 1:**
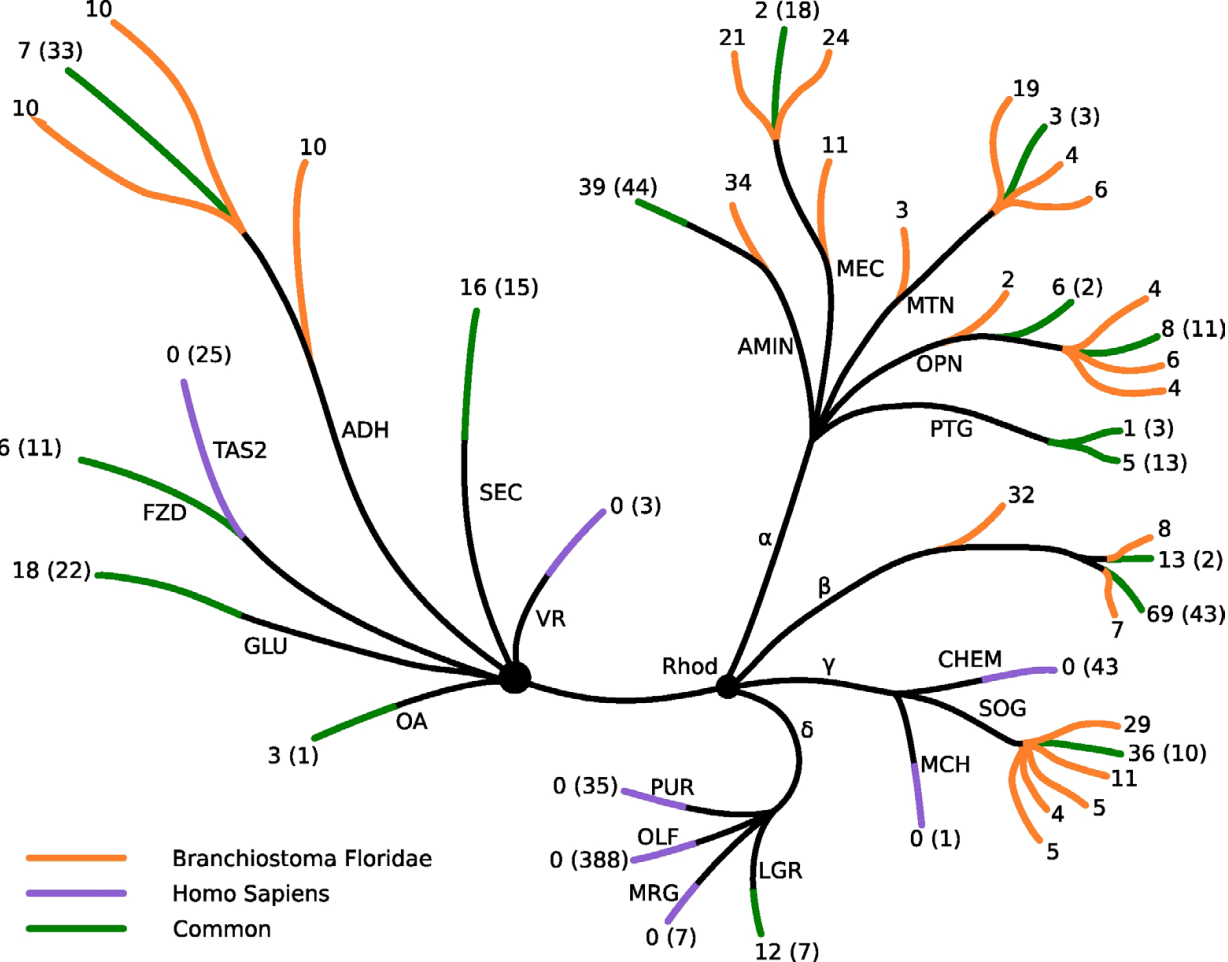
The figure summarizes our findings in amphioxus in comparison with human GPCRs. The shape of the tree and the division are adopted from [4] and the black dots represent core nodes. The families are GLU, *Glutamate*; Rhod, *Rhodopsin*; ADH, *Adhesion*; FZD/TAS2, *Frizzled/Taste2*; SEC, *Secretin*; OA, Ocular albinism receptors; VR, Vomeronasal receptors. The *Rhodopsin *family is further split into α-, β- γ- and δ-groups. The subgroups in α are AMIN, serotonin/dopamine/adrenergic/trace amine receptors; MEC, melanocortin/Lysophospholipid (EDG)/adenosin/cannabinoid receptors; MTN, Melanotonin receptors; OPN, opsin-like receptors; PTG, prostaglandin receptors. In γ: CHEM, Chemokine-like receptors; MCH, Melanocyte concentrating hormone receptors; SOG, somatostatin/opioid/galanin receptors. In δ: LGR, glycoprotein binding receptors; MRG, MAS-related receptors; OLF, olfactory receptors; PUR, purine-like receptors. Orange leafs only hold amphioxus transcripts, purple only human and green leaves represent branches that hold genes from both species. The annotation at the end of each leaf is the number of GPCRs in each branch. The number without parenthesis is for amphioxus and that one within parenthesis is the number of human GPCRs according to [13].

We identified at least 664 unique GPCRs using our highly diversified seeding datasets. Most of these are found in the main families [3]; *Glutamate (18)*, *Rhodopsin (570)*, *Adhesion (37)*, *Frizzled (6) *and *Secretin (16) *(see Fig. 1). We did, however, not find any bitter taste (Taste 2) or vomeronasal (VR1) receptor. These types of receptors are abundant in rodents and most likely all other non-primate mammals but are rare in fish and chicken [8]. Neither did we find evidence for any members of the many pre-vertebrate lineage-specific expansions such as the nematode chemosensory receptors, the gustatory receptors from insects, the odorant receptors from *Drosophila melanogaster*, MLO receptors in plants or fungal pheromone (STE2 and STE3) from yeast [4,12]. We found, however, two sequences that show some similarity with the cAMP-binding receptors from slime moulds but the identity is fairly low (25.6%) and further work needs to determine if they are true cAMP receptors.

**Figure 2:**
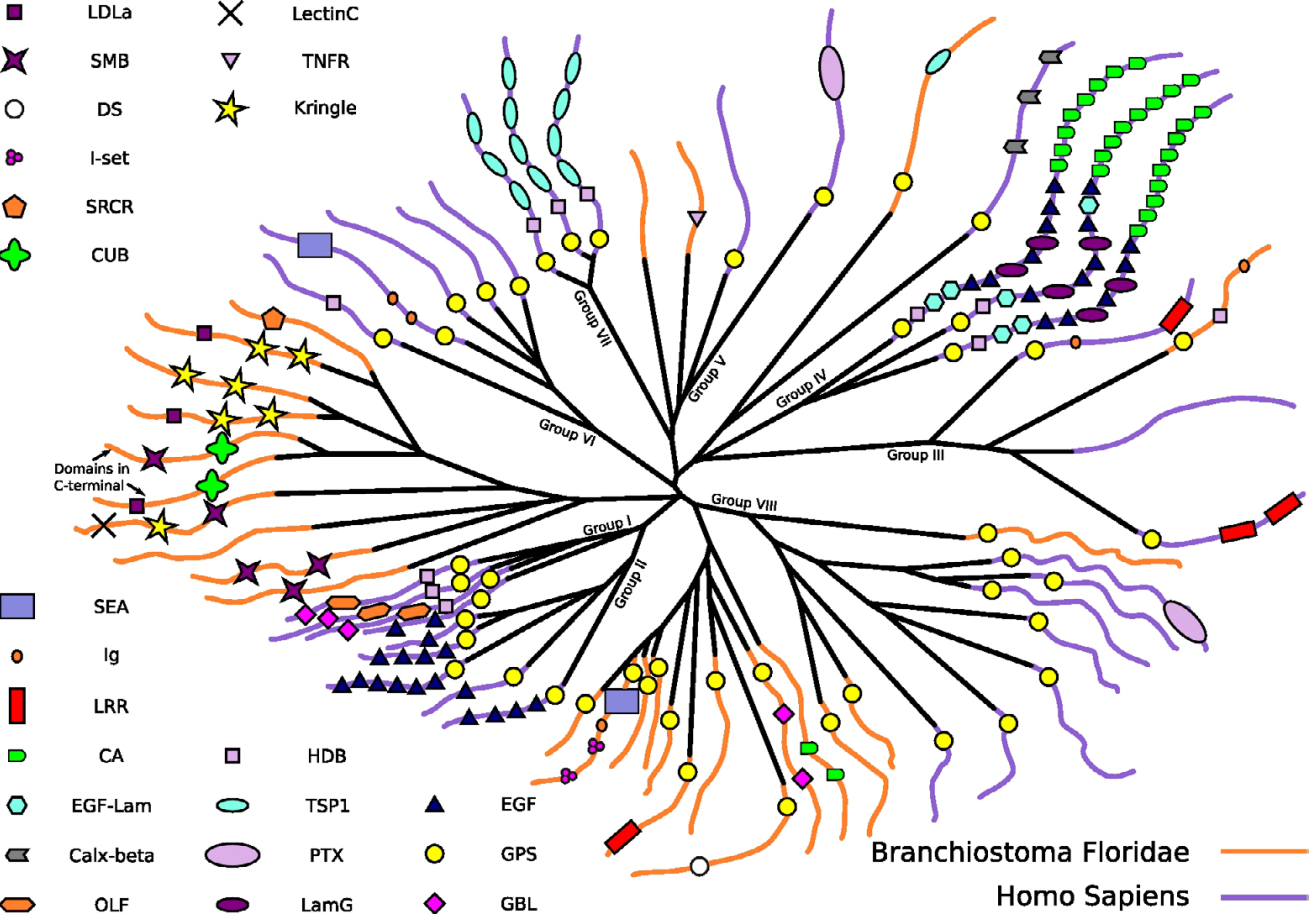
Schematic picture of the phylogenetic relationship and the functional domains within the *Adhesion *GPCR family in amphioxus and human. The tree is based on a neighbor-joining tree bootstrapped 500 times. The colours of the leaves denote the species; orange for amphioxus and purple for human genes. The leaves also show the domains of the N-termini, except in two explicitly marked cases were it is C-termini that contain the domains. The domain search in amphioxus was made with the HMMER package, an e-value cut-off at 0.01 and the local PFAM models. Human domains were adopted, along with group annotations, from [13]. The symbols and abbreviations are shown in the lower left corner. The abbreviations stand for: GPS, GPCR proteolytic site; EGF, epidermal growth factor; HBD, hormone-binding domain; Ig, immunoglobulin; OLF, olfactomedin; GBL, galactose-binding lectin domain; CA, cadherin domains; LamG, laminin; LRR, leucine rich repeats; SEA, sperm protein, enterokinase, and agrin; TSP, thrombospondin; PTX, pentraxin domain. In the upper left corner are the domains unique for amphioxus. The abbreviations are: LDLa, Low-density lipoprotein receptor domain class A; SMB, Somatomedin B domain; DS, discoidin; I-set, Immunoglobulin I-set domain; CUB, CUB domain; LectinC, Lectin C-type domain; TNFR, TNFR/NGFR cysteine-rich region; Kringle, Kringle domain; SRCR, Scavenger receptor cysteine-rich domain.

The *Adhesion *family of GPCRs has vague similarity with the *Secretin *receptors in the transmembrane (TM) regions but their structural and functional properties are very different [3]. There are 33 known *Adhesion *receptors in human and these have characteristic long N-termini containing multiple functional domains [13,14], known to be present in other protein families. Interestingly, we found a very rich repertoire of *Adhesion *GPCRs, in total 37, in amphioxus(see Fig. 2 and Additional file 1, figure S1). Some of the mammalian groups [8,13] are missing, such as the groups I (lectomedin receptors) and II (epidermal growth factor module-containing receptors and cell differentiating antigen receptor) that is likely to have evolved late in the vertebrate lineage [15]. Three of the *Adhesion *groups have both mammalian and amphioxus members. These are group III (orphans, expressed in CNS), V (orphans) and VIII (orphans with highly variable N-terminal length). One orthologous receptor to the very long G protein-coupled receptor 1 (VLGR1) is also present. The remaining missing groups are group IV (CELSR with multiple cadherin domains), VI (GPR110, GPR111, GPR113, GPR115 and GPR116) and VII (brain-specific angiogenesis inhibitor receptors). Many of the amphioxus *Adhesion *GPCRs have the GPS domain, which are characteristic for the *Adhesion *family and not found in any other GPCR family including the *Secretin *receptors. Moreover, many of the amphioxus *Adhesion *GPCRs have multiple domains in the N-termini, which is another important feature of the mammalian *Adhesion *GPCRs. These N-termini are likely to mediate cell-to-cell interaction which for instance could allow participation in different types of cell guidance [16]. Surprisingly, we found several novel domains in the N-termini of the amphioxus *Adhesion *GPCRs like Somatomedin B, Kringle, Lectin C-type and SRCR (for more details see Fig. 2) which to our knowledge are unique for amphioxus among the GPCRs. Also the domains LDLa, Immunoglobulin I-set, CUB and TNFR were found and they can not be found in mammal *Adhesion *GPCRs [13]. Although; especially interesting is the Kringle and Somatomedin B domains which are found in sequences from a previously uncharacterized *Adhesion *expansion of ten genes (Fig 2). The Kringle domain (see alignment in Additional file 2, figure S2) is a protein-binding domain [17] present in urokinase-type plasminogen activator (uPA) while the Somatomedin B domain (Additional file 2, figure S1) can be found in vitronectin [18]. These two proteins interact and the Somatomedin B domain and helps in the localization of uPA to focal adhesions in microvessel endothelial cells. Interestingly, all of these domains not previously identified in *Adhesion *GPCRs, have a large number of conserved cysteines which is a feature consistent with many other common domains found in this family. This similarity could suggest that these domains are inserted through domain shuffling of similar stretches of DNA. Interestingly, many of these new domains have recognised cell adhesion properties and participate in cell guidance. The genes in the other cluster in this family share a resemblance to invertebrate genes according to the top five hits in BLAST searches against the NCBIs (National Center for Biotechnology Information) non-redundant (nr) database. These invertebrate genes are primarily from either *Strongylocentrotus purpuratus *which is, according to Delsuc et al., closely related to amphioxus [10] or, more interestingly, from the more distantly related cnidarian *Nematostella vectensis*. In average 4.4 of these first five hits are from invertebrate species (see Additional file 3). These findings suggest that the *Adhesion *family, with its unique structural and functional characteristics, has a very long evolutionary history and that these are likely to be present in most vertebrates. Moreover, these properties seem to have undergone further diversification within the lineage leading to amphioxus, and are likely to have gained additional roles in cell guidance.

The *Glutamate *family of GPCRs, also known as family C, have previously been divided into eight groups with the largest, the metabotropic glutamate receptors (mGluRs) containing eight members in humans. In addition, the *Glutamate *family contains GABA (gamma amino butyric acid) B receptor, the calcium sensing receptor (CASR) and the related amino acid binding GPRC6A, taste receptors for sweet and umami (TAS1Rs), the orphan GPR158 as well as GPRC5 receptors, a group of four related orphan receptors. Moreover, the *Glutamate *family in mammals contain the large family of pheromone receptors designated vomeronasal receptors type 2 (V2Rs) which in the mouse genome contains over 50 functional genes while all V2Rs in the human genome are pseudogenes [19]. In amphioxus, we identified 17 receptor sequences from the *Glutamate *family (Additional file 1, figure S4) while the human genome contains 22 sequences [19]. All the amphioxus sequences except three are most similar to vertebrate sequences according to a BLAST search against NCBIs nr dataset (see Additional file 3). Two of the invertebrate-like sequences hit insect related genes while the third hits genes from the invertebrates *Tribolium castaneum*, *S. purpuratus*, *Apis mellifera*, *N. vectensis *and the vertebrate *Tetraodon nigroviridis*. Of the eight mammalian groups, four are represented in amphioxus while the branches of TAS1Rs, CASR, GPRC6A and V2Rs are missing. TAS1R, GPRC6A, and V2Rs were previously found to be present in the teleost *Danio rerio *and missing in *C. intestinalis *[19] and our findings here support that these receptor groups are specific for vertebrate species. Curiously, we did not find the gene for CASR in amphioxus which has previously been observed in *Caenorhabditis elegans*, indicating that this receptor was lost specifically in the lineage leading to amphioxus.

Amphioxus has also a characteristic *Secretin *family of GPCRs with distinctive features of a hormone binding domain in the N-termini. In line with all known vertebrate species, amphioxus does not have any of the methuselah receptors [20], which are related to the *Secretin *receptors and found in arthropods. The presence of typical *Secretin *GPCRs in amphioxus provides further evidence that *Secretin *and *Adhesion *are separate evolutionary branches in vertebrates. The *Secretin *family can be divided into five main branches a) Corticotrophin Releasing Factor (CRF); b) Secretin (SCT), Vasoactive Intestinal Peptide (VIP), Pituitary Adenylate Cyclase-Activating Polypeptide (PACAP) and Growth Hormone Releasing Hormone (GHRH); c) Glucagon (GCG), Glucagon-Like Peptide (GLP), Glucose Insulinotropic Peptide (GIP); d) Parathyroid Hormone (PTH) and e) Calcitonin (CAL) and Calcitonin Gene-Related Peptide (CGRP) [21,22] of which groups a, d and e are found in amphioxus (see Additional file 1, figure S5). Recent studies on the *Secretin *family shows that the receptors in group a and e are ancient and found in both deuterostoma and protostoma [23], but the sequences we identified in amphioxus all show a closer relationship with vertebrates (see Additional file 3). These branches, together with group d, are also present in amphioxus while we do not find any receptors from group b and c in amphioxus. The missing groups contain the *Secretin *GPCRs binding gut peptides in mammals, suggesting that these functions are missing in amphioxus (see Additional file 4, table S2). On the other hand, several of the *Secretin *receptors (group a and e) that mediate hypothalamus/pituitary signalling in mammals are present (see Additional file 4, table S1). This may suggest that parts of the gut functions found in vertebrates are missing or are regulated differently in amphioxus compared to vertebrates. However, there are good evidence that amphioxus has GPCRs that are likely to participate in endocrine functions that may resemble that of hypothalamus and pituitary in mammals [24], such as corticotropin releasing hormone receptor, somatostatin receptors and vasopressin/oxytocin receptors (Additional file 4, table S1). It is also notable that several GPCRs known to participate in pancreatic functions are missing, such as cholecystokinin receptor A and B, gastric-inhibitory peptide receptor and the secretin receptor (Additional file 4, table S2). However, insulin like peptides [25] and receptors [26] have been shown to be present suggesting presence of some of the most fundamental pancreatic functions.

The *Frizzled *family contains ten frizzled and the smoothened receptor in most mammals and are the most well conserved of all GPCR families both regarding family members and primary amino acid sequence [8]. *Frizzled *receptors are found in all bilateral species and are crucial for early embryonic development while being the receptor for Wnt and Sonic hedgehog. Frizzled-like receptors have also been found in the unicellular slime mold *D. discodieum *which was hypothesized to carry 25 frizzled genes in its genome [5], although these clearly have a borderline similarity to mammalian *Frizzled *GPCRs [6]. We found five *Frizzled *receptors as well as an ortholog to smoothened in amphioxus (Additional file 1, figure S2). These are all vertebrate like according to their five best hit against NCBIs nr dataset. This is in line with our previous study indicating that vertebrates, including teleost fish, have a repertoire similar to that of humans, while invertebrates have fewer; between 5 (*C. elegans*) and 7 (*D. melanogaster, Anopheles gambiae *and *C. intestinalis*) *Frizzled *GPCRs [4]. The increased number of *Frizzled *GPCRs probably reflects a requirement for higher signal diversity for formation of the more complex body plan and, in particular, the more complex nervous system of vertebrates.

**Figure 3:**
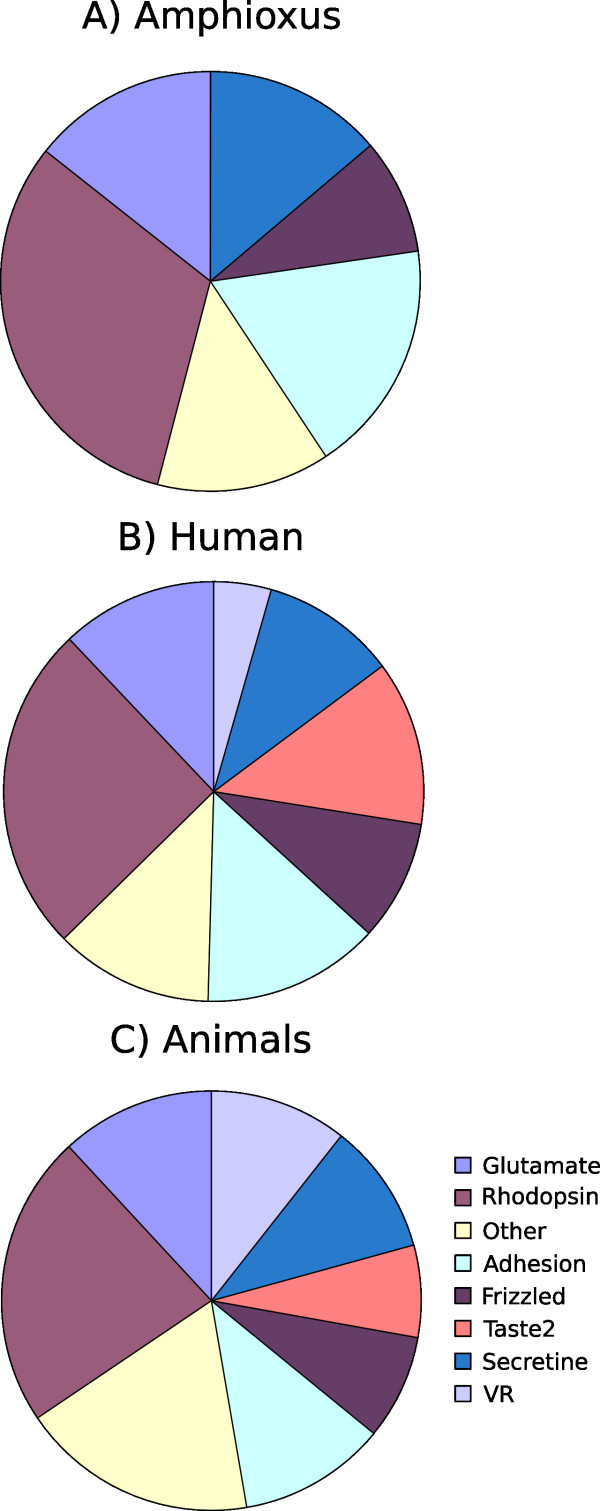
Distribution of the number of different GPCR families in A) amphioxus, B) Humans and C) All animals, displayed on a logarithmic scale. The Animal diagram is based on an average for amphioxus, humans, *Mus musculus*, *Gallus gallus*, *Danio rerio*, *Takifugu rubripes*, *Ciona intestinalis*, *Drosophila melanogaster*, *Anopheles gambiae *and *Caenorhabditis elegans*. The numbers for humans and *M. musculus *are from [13], *G. gallus *from [8] and for the remaining species from [4].

The *Rhodopsin *family of GPCRs constitutes the largest family of GPCRs in vertebrates (Fig. 3). The largest group of *Rhodopsin *GPCRs in mammals are the mammalian type of olfactory receptors, comprising 388 members in humans. This type of olfactory receptors are also found in chicken [8] while there are markedly fewer in fish such as *D. rerio *and *Takifugu rubripes *[4] but we can not identify these in amphioxus. The *Rhodopsin *GPCRs have been divided into four main groups, termed α-,β-, γ- and δ-group [3], which in turn could be further subdivided into thirteen subgroups. Like most vertebrates, the *Rhodopsin *family in amphioxus contains the largest number of GPCRs (Fig 1). These GPCRs are found in all four main groups of *Rhodopsin *GPCRs. However, only eight of the thirteen subgroups are present in amphioxus (See Additional file 1, figure S6-S16); missing are the mammalian type of olfactory receptors, the chemokine, the melanin concentrating hormone (MCH), the MAS-related and the purin receptor subgroup. This is in line with our previous study indicating that MAS-related receptors are missing in teleost fish and that *C. intestinalis *is missing four of these five groups [4]. The fact that *C. intestinalis *has a chemokine receptor cluster strengthens the argumentation forwarded by Delsuc et al. that tunicates and not cephalochordates are the closest living relatives to the vertebrates [10].

One dopamine receptor, AmphiD1/β, has been identified in amphioxus [27,28]. Recently, 11 receptors related to the large cluster of biogenic amine receptors were identified in amphioxus from unassembled genomic sequence reads and shown to have tissue specific expression [29]. Our searches from the assembled genome identified additional 33 receptors belonging to the amine receptor cluster. Of the previously known receptors AmphiAmR3 and R7 cluster with histamine receptor 4 in our phylogenetic tree, AmphiAmR1 clusters with dopamine receptors D1 and D5, AmphiAmR4, R5, R6, R9 and R11 is found in a non-resolved cluster with only amphioxus sequences while we placed AmphiD1/β basal to the amine subgroup as it could not find a stable location in the tree. AmphiAmR2, R8 and R10 placed basal in the *Rhodopsin *family. Of the new amphioxus receptors we identified, 18 fall in groups containing only amphioxus sequences in the phylogenetic tree (Additional file 1, figure S6), indicating that they are not clearly orthologous to any of the mammalian receptors. However, we also identified receptors that fall into specific groups of human receptors, suggesting the possibility that they are sharing ligands with the orthologous human receptors. We identified receptors that place in the D2/D3 dopamine cluster, the muscarinic receptor cluster and the adenosine receptor cluster. Moreover, we found orthologs to three serotonin receptors, namely 1, 5 and 6 as well as six receptors that place close to histamine receptor 4 (Additional file 1, figure S6). In addition to the rich set of amine receptors, amphioxus has large set of peptide binding receptors and predicted peptide ligands, including several receptors (neuropeptide Y, neuropeptide FF, uterinbombesin, neuromedin and growth hormone secretagogues receptors) with ligands involved in appetite control (see further details in Additional file 4, table S1-S3).

The *Rhodopsin *family in amphioxus contains several larger expansions of GPCRs (see Additional file 1, figure S6-S16). The largest of these includes the Neuropeptide FF receptor in the β-group (39 sequences), somatostatin, opioid, galanin cluster (29 sequences), two expansions in the melanocortin, endothelin cannabinoid clusters with 21 and 24 sequences each and one in the melatonin receptor cluster (19). The β-group, which has several peptide receptors, also has a large expansion of twenty predicted genes that are similar to the prokineticin receptor (PK) 1 and 2 which are important for contraction of gastrointestinal smooth muscles [30]. These are, on average, about 42 percent identical in the TM regions with PK1 and PK2 receptors. This expansion is surprising, however as prokineticin is a very potent muscle contractor, it is possible that these receptors are involved in these early specific pre-vertebrate muscles that create effective undulations of the amphioxus body.

163 amphioxus sequences had more resemblance to invertebrate sequences than to vertebrate transcripts according to the BLAST searches. Beside the expansion in the *Adhesion *family, most of these are categorized to the *Rhodopsin *family (111) or these are sequences we could not categorize (28). Still, none of the larger *Rhodopsin *clusters mentioned above show similarity to many invertebrate like sequences with exception of the melatonin cluster which shows similarity to several *S. purpuratus *genes. There is also a cluster in the β-group of the *Rhodopsin *family holding seven transcripts that all are insect like and have all hits related to the ecdysis triggering hormone receptor [31].

We found several examples of support for Ohno's 2R hypothesis that postulates that two whole genome duplication occurred in early vertebrate evolution [32,33]. The phylogenetic trees show that there are several branches with multiple human members where amphioxus sequences place basal (closer to the root of the tree) on the branch. For example, the tachykinin receptors 1–3 and frizzled receptor 1,2 and 7 both cluster with a single amphioxus sequence with high bootstrap values. These belong to paralogon groups (PG) 9 (Meta HOX) [34,35] and 10 (HOX paralogon), respectively. Moreover, the muscarine receptors 1,3,5 in PG4 have basal amphioxustranscripts as well as adenosin binding receptors 1, 2A, 2B in PG11, brain specific angiogenesis-inhibitory receptors 1–3 in PG14 and somatostatin receptors 2,3,5 in PG12.

## Conclusion

Overall, the amphioxus genome is in general terms surprisingly rich in GPCRs, with extensive representation of receptors from all of the main mammalian GPCRs families (See Fig 1). The repertoire resembles that of vertebrates but lacks the branches previously known to be vertebrate-specific. The groups that are missing include the chemokine receptors which are important for the immune system and receptors for purines which serve as important co-transmitters within the nervous systems. Amphioxus lacks many of the classical mammalian sensory receptors such as the pheromone receptors that are particularly important for non-primate mammals and also the mammalian olfactory receptors. Moreover, amphioxus does not have the bitter taste receptors (TAS2) or the sweet and umami (TAS1) receptors which suggest that amphioxus have a unique way of detecting these taste modalities. The amphioxus genome does not have any other classical GPCR group that recognizes exogenous stimuli, such as mammalian type olfactory receptors or pheromone receptors. On the other hand, amphioxus has opsin receptors, showing conservation in the visual system. It should however be mentioned that sensory GPCRs evolve rapidly and there are many species specific expansion of such receptors. It can thus not be excluded that there are sensory receptors that are not found in this type of searches. Amphioxus has fewer receptors that clearly can be recognised as monoamine receptors (only 1 muscarinic, 2 dopamine, 3 serotonin and one histamine) which are involved in neurotransmission in higher animals. Amphioxus does, however, have a large number of other receptors with high similarity to amine and peptide receptors that suggest early development of these systems that are very important for CNS functions.

## Methods

### Search & confirmation

The amphioxus genome and protein sequences were downloaded from US Department of Energy Joint Genome Institute [11]. Protein transcripts shorter than 250 amino acids were removed as they are too short to hold a 7TM-region. The remaining amphioxus proteins were searched with GPCR hmm models adapted from [4] using hmmer [36], which is available from [37]. All hits with an E-value below 0.01 were aligned with BLAST [38] against a database consisting of the human RefSeq [39] together with nematode chemoreceptors, the gustatory receptors from insects, the odorant receptors from *D. melanogaster*, MLO receptors in plants [12], fungal pheromone receptors (STE2 and STE3) from yeast and cAMP receptors from *D. discodieum*. For each putative amphioxus GPCR, the top five hits were inspected and if a minimum of four of these were GPCRs, the transcript was accepted as a GPCR. Alternative splice variants were removed by aligning the amphioxus proteins to their genome with BLAT [40]. As the amphioxus genome has not yet been assembled and only consists of scaffolds this method does not catch all splice-variants and duplicates.

### Categorization

In order to categorize the amphioxus GPCRs, human and other GPCRs were tagged with their family name. The human receptors were divided into the GRAFS families [4], *Glutamate *(GLU), *Rhodopsin *(Rhod), *Adhesion *(ADH), *Frizzled *(FZD) and *Secretin *(SEC). The Taste2 receptors were separated from *Frizzled *receptors. In addition, the *Rhodopsin *family was split in accordance to [4]. The other GPCR families used in this step were nematode chemosensory receptors, the gustatory receptors from insects, the odorant receptors from *D. melanogaster*, MLO receptors from plants, fungal pheromone receptors (STE2 and STE3) from yeast and cAMP receptors from *D. discodieum*. The putative amphioxus GPCRs were aligned against a database consisting of these tagged GPCRs and had to have at least four of the five best hits from the same family to be assigned to this family. A multiple alignment was made for each family and the alignments were viewed in Jalview [41], and the amphioxus sequences were cut with the reference transcripts as a model to gain only the 7TM regions. This process was repeated three times in order to only remove sections in inverse proportion to how well they aligned, balanced against the alignment quality. First, the N and C terminals were removed, except for about twenty amino acids in each end. Second, internal loops were removed and in the last round N and C terminals were trimmed to exactly match the models. In order to remove duplicate sequences, all the cut amphioxus sequences were aligned against each other, using BLAT, and all pairs with identical sequences were removed. Maximum-likelihood trees were also created with PHYLIP and viewed in MEGA [42]. All sequences in a family were aligned to each other and an identity distance matrix was constructed using Megalign from the DNASTAR package (DNASTAR, Madison, Wisconsin, United States). Amphioxus sequences with identities below ten percent towards their reference were discarded as random hits. If the identity was between ten and twenty, they were considered basal to the family. We calculated a neighbor-joining tree, bootstrapped five hundred times, using MEGA with default parameters based on an alignment made in MEGA using the ClustalW algorithm and batch ClustalW default parameters for the sequences with identities above twenty percent to at least one of the reference sequences. Sequences disrupting the tree were moved to the basal group on the basis of low bootstrap values and poor alignments through manual inspection. Additional identity checks were made with the EMBOSS [43] implementation of the Smith-Waterman local alignment algorithm. Domain search was performed with the PFAM domain dataset and the HMMER package.

## Authors' contributions

KN carried out the study, participated in the design and drafted the manuscript. RF and HS conceived the study, participated in its design and in the writing of the manuscript. All authors read and approved the final manuscript.

## Supplementary Material

Additional file 1 Neighbor-joining trees. Phylogenetic trees of the amphioxus GPCRs. Click here for file

Additional file 2 Multiple alignments of the Somatomedin B and the Kringle domains. Two multiple alignments of the Somatomedin B and the Kringle domains found in an expansion of the amphioxus *Adhesion *family. Click here for file

Additional file 3 BLAST search against the nr database. Results from the BLAST search of the amphioxus GPCRs against NCBIs nr database. The top five hits, excluding hits from the *Branchiostoma*-phylum, are presented. Click here for file

Additional file 4 Amphioxus neuropeptide, gut peptide and food intake receptors orthologs. Compilation of the orthologs to known vertebrate neuropeptide, gut peptide and food intake receptors. Click here for file
