# Supplementary material for: The amphioxus (Branchiostoma floridae) genome contains a highly diversified set of G protein-coupled receptors
Source: BMC Evol Biol. 2008 Jan 16;8:9. doi: 10.1186/1471-2148-8-9 (PMC2246102; doi:10.1186/1471-2148-8-9)
Supplement: Additional file 1 — Neighbor-joining trees. Phylogenetic trees of the amphioxus GPCRs. [file 1471-2148-8-9-S1.pdf]

Additional file 1– Neighbor-joining trees

All trees were made with MEGA using sequences cut with the TM-domain from the reference set as model. The sequences were aligned with the ClustalW-algorithm and the standard parameters used in the batch mode variant. The trees are neighbor-joining trees that were bootstrapped 500 times. The bootstrap values in the trees are given in percent. The annotation after the sequence name contains information about the sequence, such as doublets and domain hits. The domains are given with their Pfam id (i.e. 7tm_1 for the *Rhodopsin* family transmembrane domain). The xN (ie x2) always refers to the object closest before it. If it is directly after the name it denotes the number of copies of the sequence and if it is after a domain name it stands for the number of hits with that domain. Domain searches were made with the HMMER package and the Pfam library of local domains (Pfam_ls). An e-value cutoff at 0.01 were applied.

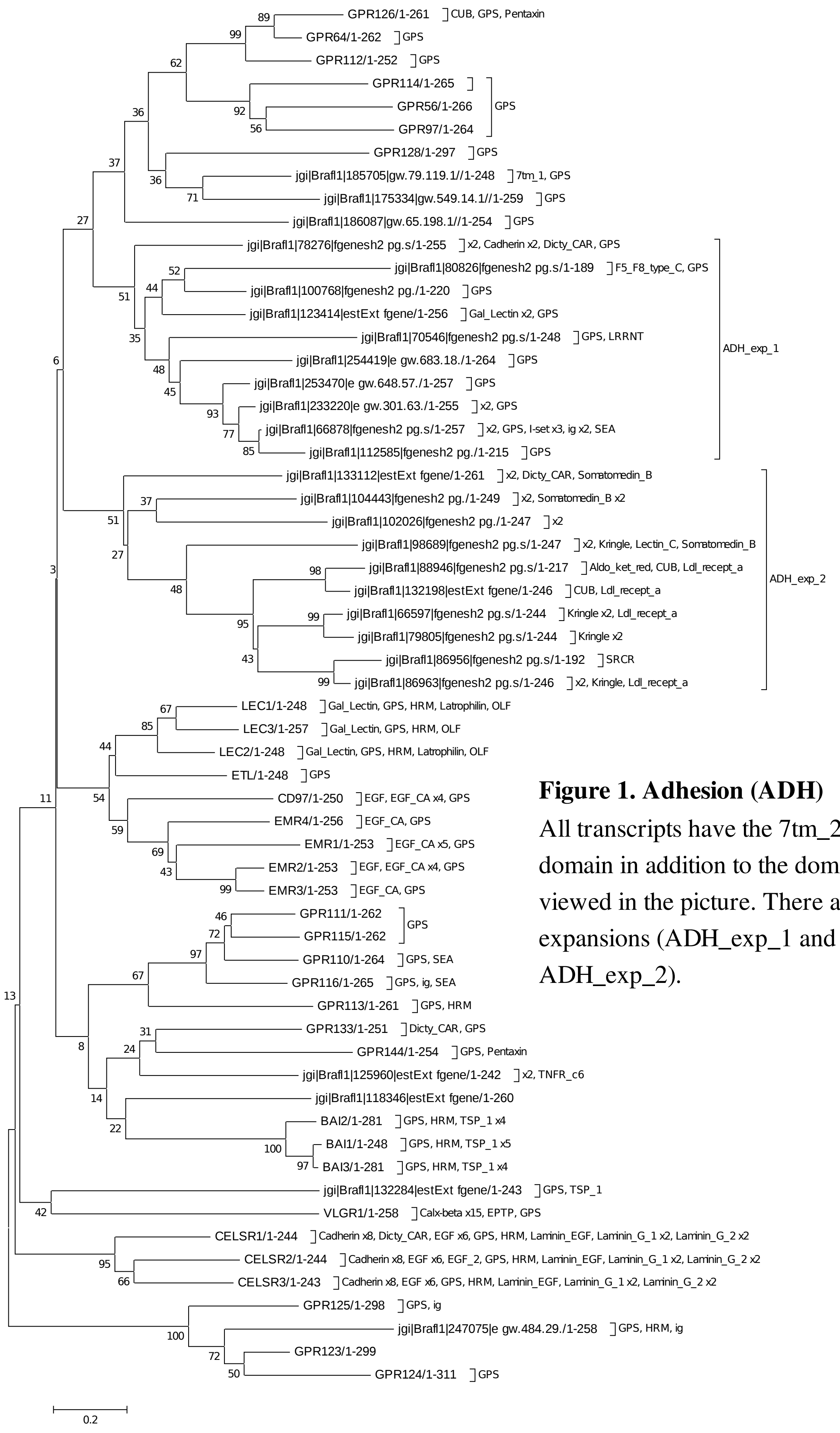

Figure 1. Adhesion (ADH) All transcripts have the 7tm_2 domain in addition to the domains viewed in the picture. There are two expansions (ADH_exp_1 and ADH_exp_2).

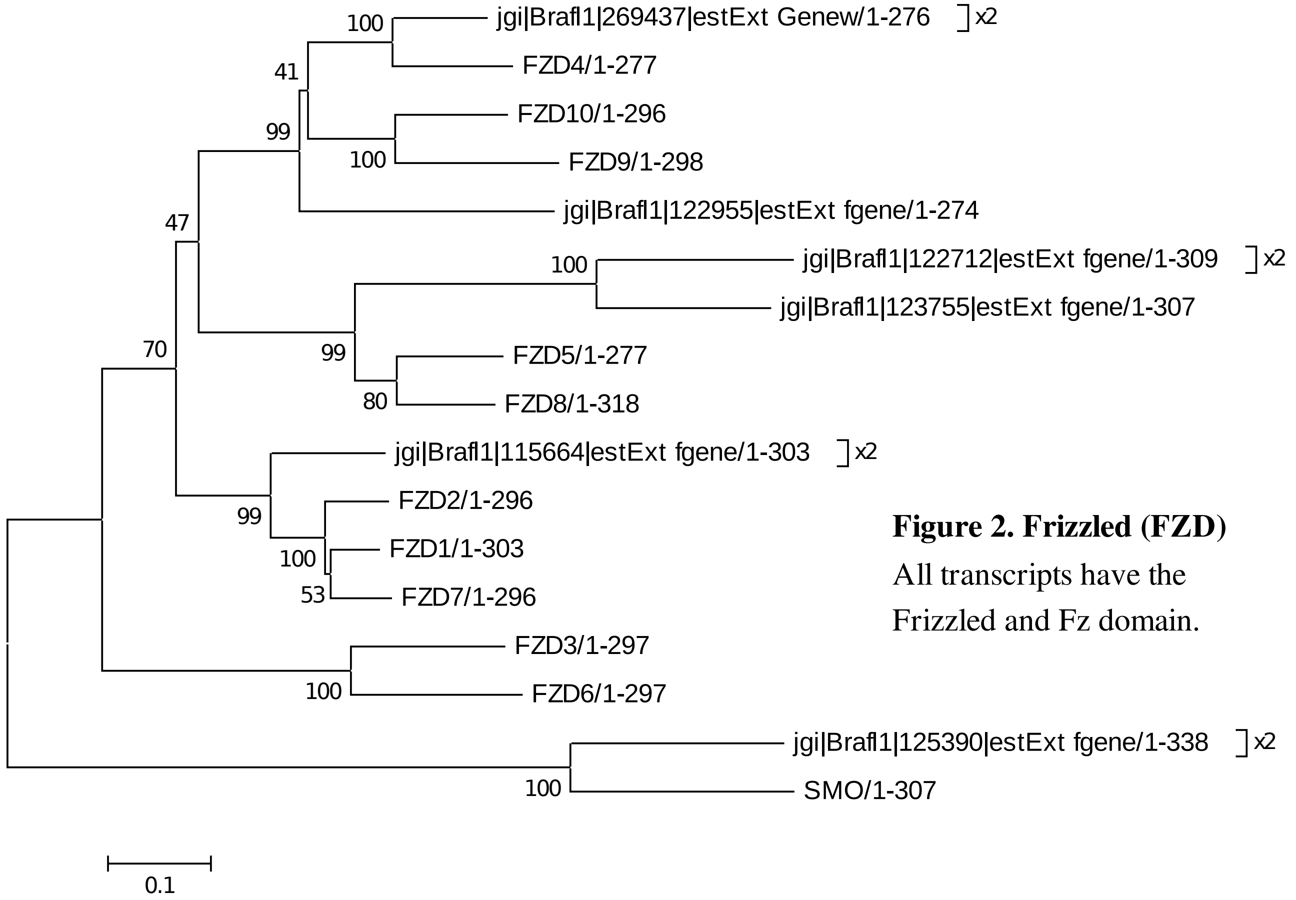

Figure 2. Frizzled (FZD)
All transcripts have the Frizzled and Fz domain.

Figure 3. Occular albinism (OA)
All transcripts have the Ocular_alb domain.

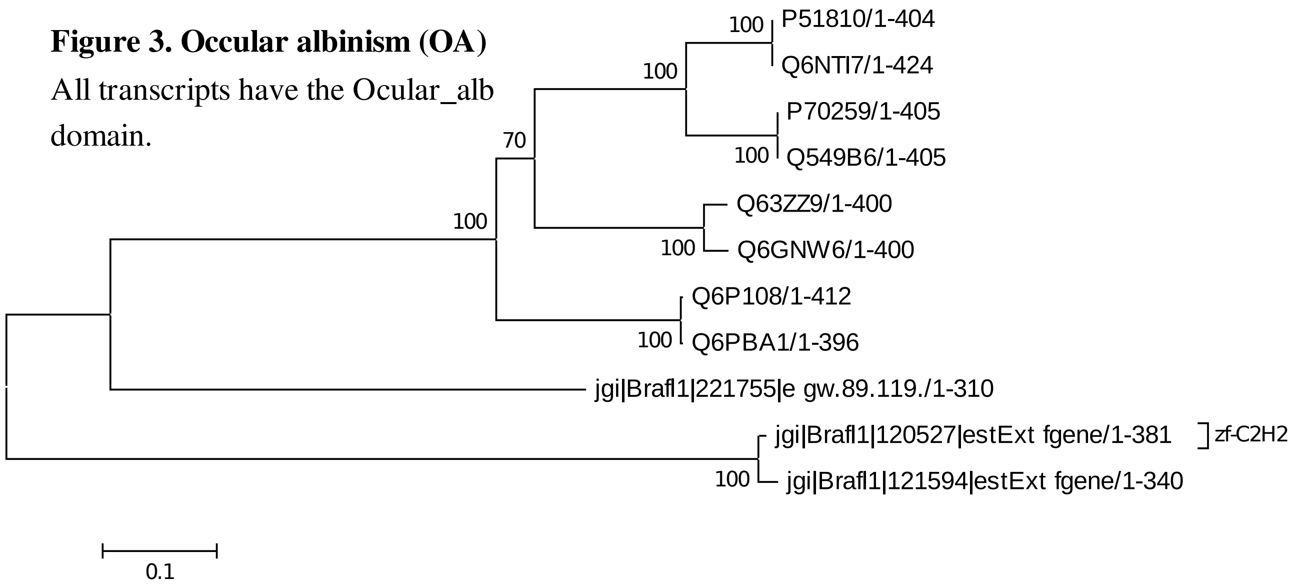

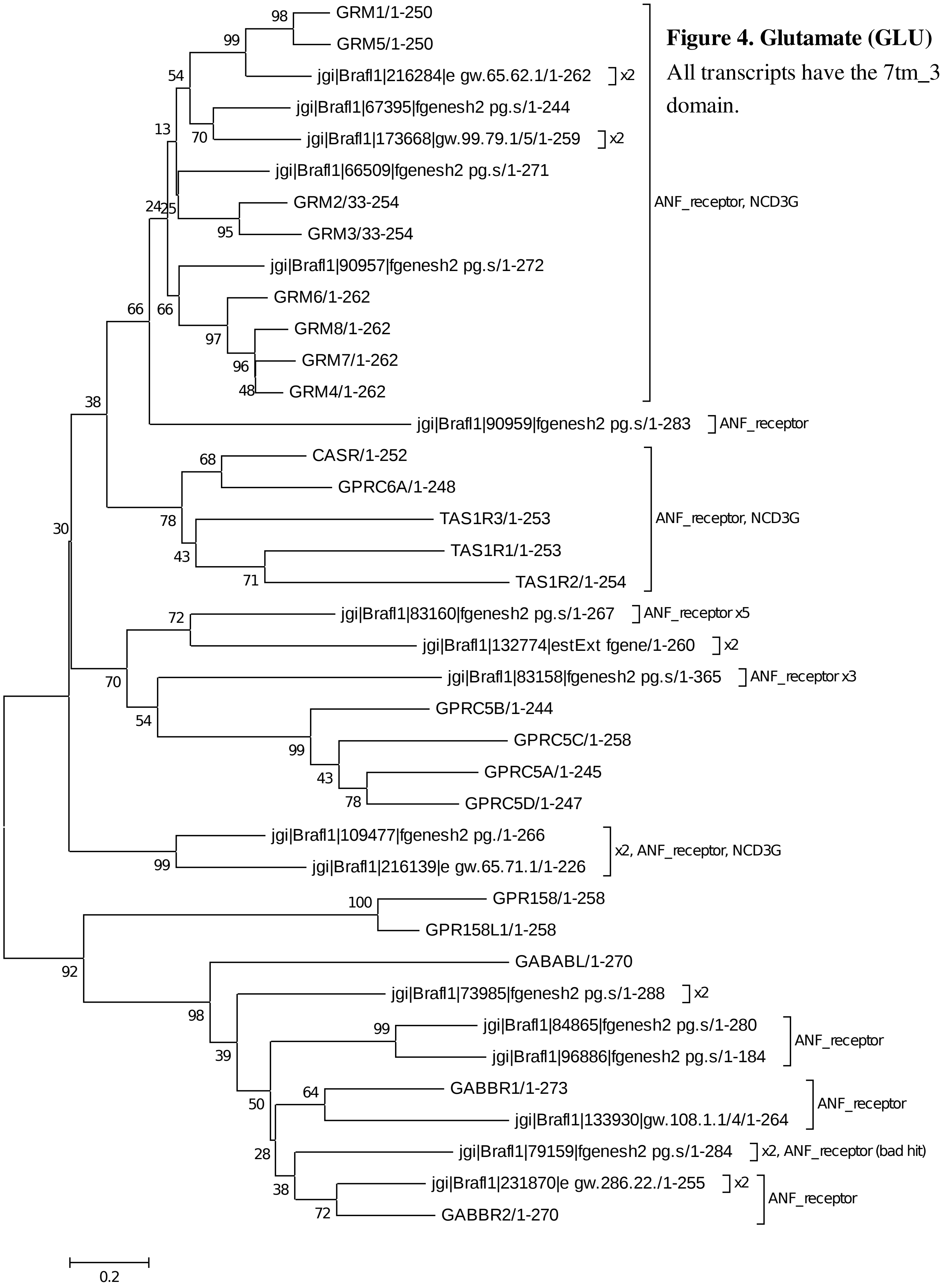

Figure 4. Glutamate (GLU)
All transcripts have the 7tm_3 domain.

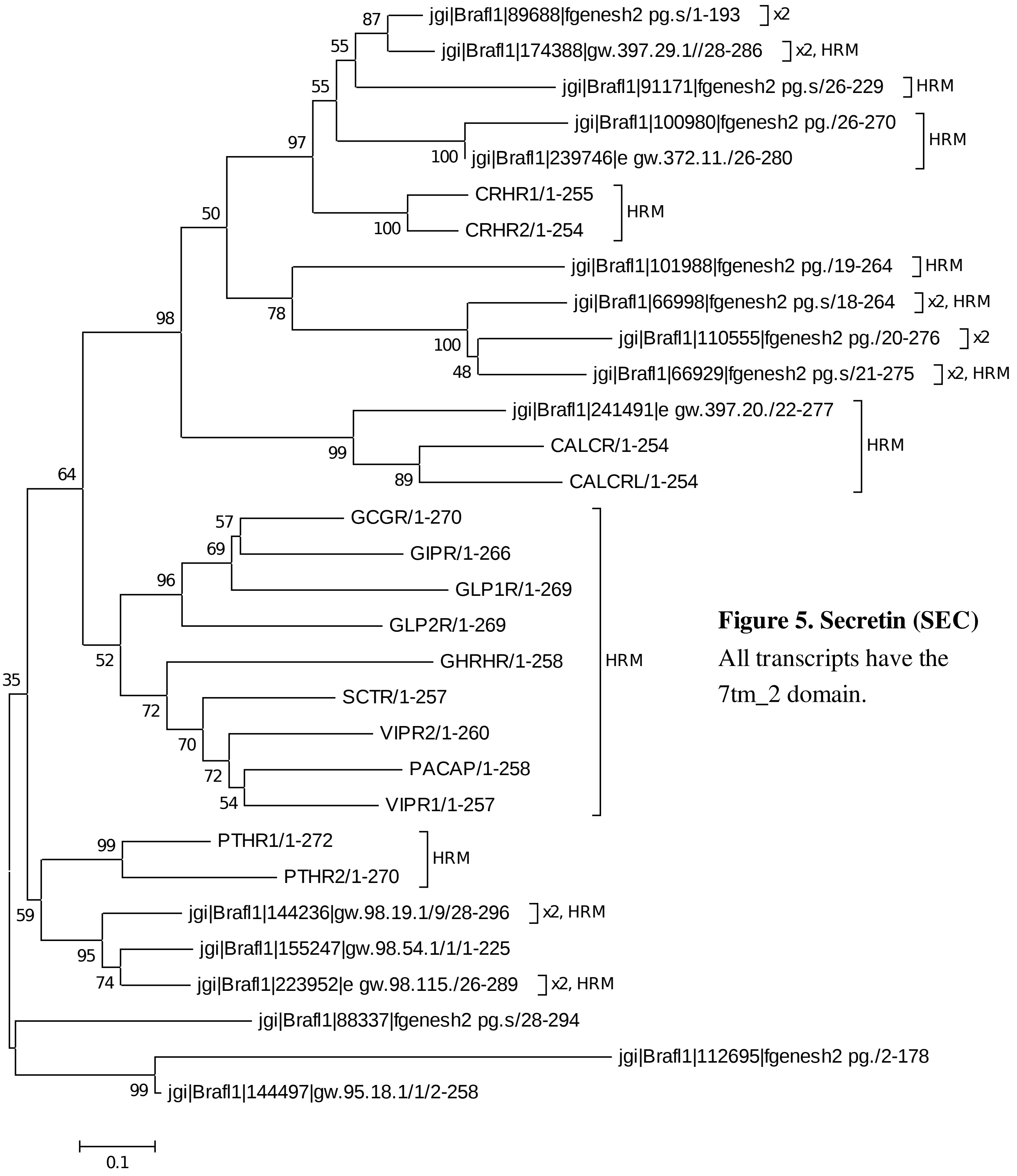

Figure 5. Secretin (SEC)
All transcripts have the 7tm_2 domain.

Rhodopsin alfa

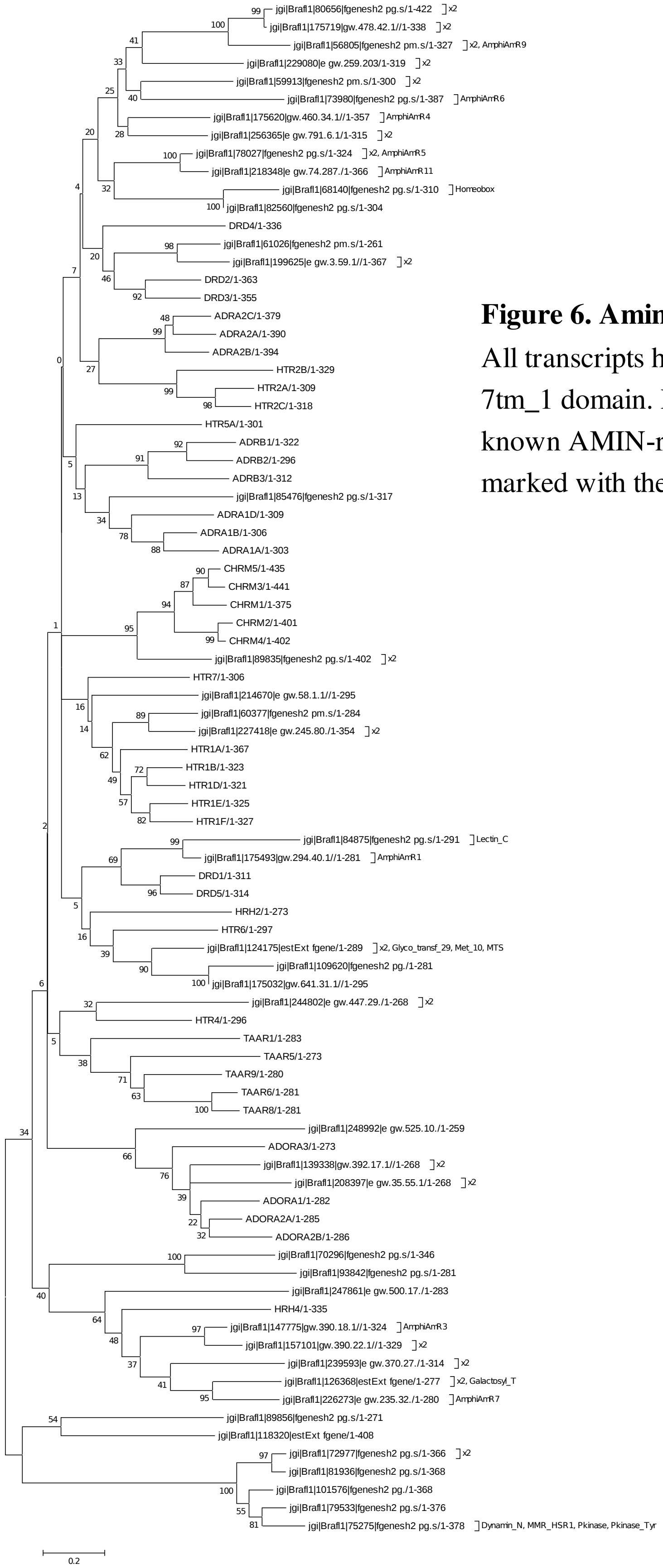

Figure 6. Amine (AMIN) All transcripts have the 7tm_1 domain. Previously known AMIN-receptors are marked with their name.

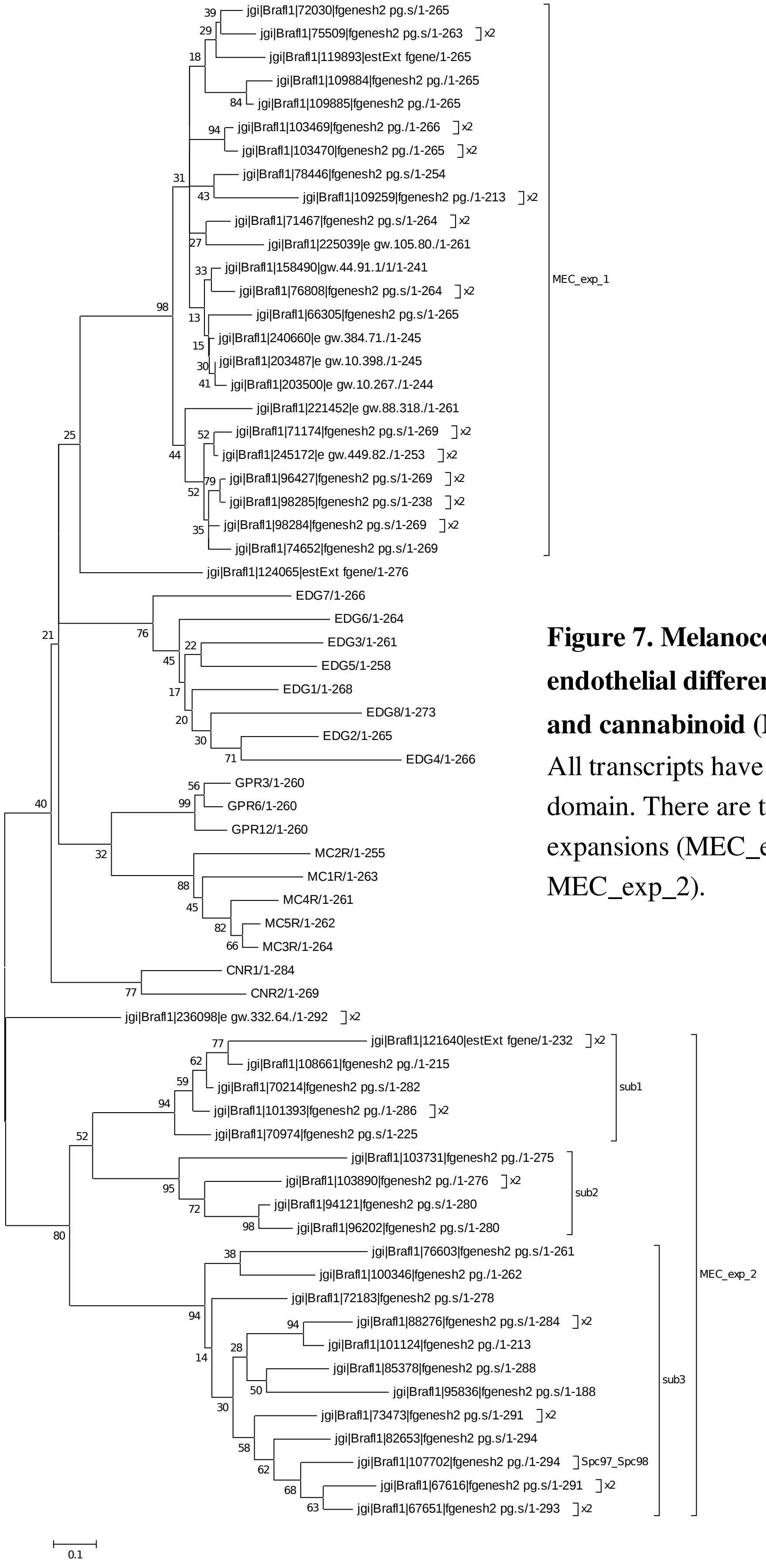

Figure 7. Melanocortin, endothelial differentiation and cannabinoid (MEC) All transcripts have the 7tm_1 domain. There are two expansions (MEC_exp_1 and MEC_exp_2).

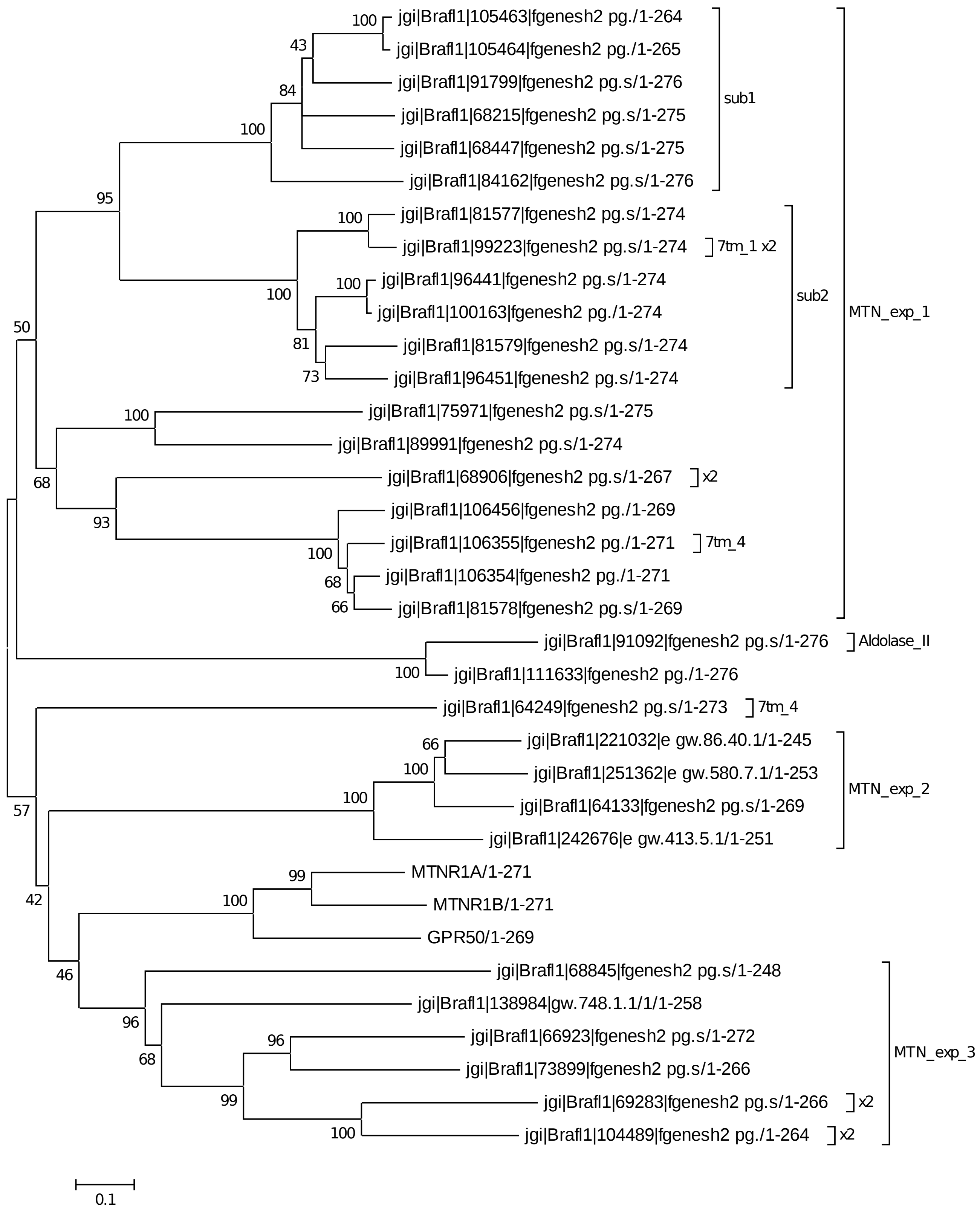

Figure 8. Melatonin (MTNR)

All transcripts have the 7tm_1 domain. There are three expansions (MTN_exp_1, MTN_exp_2 and MTN_exp_3).

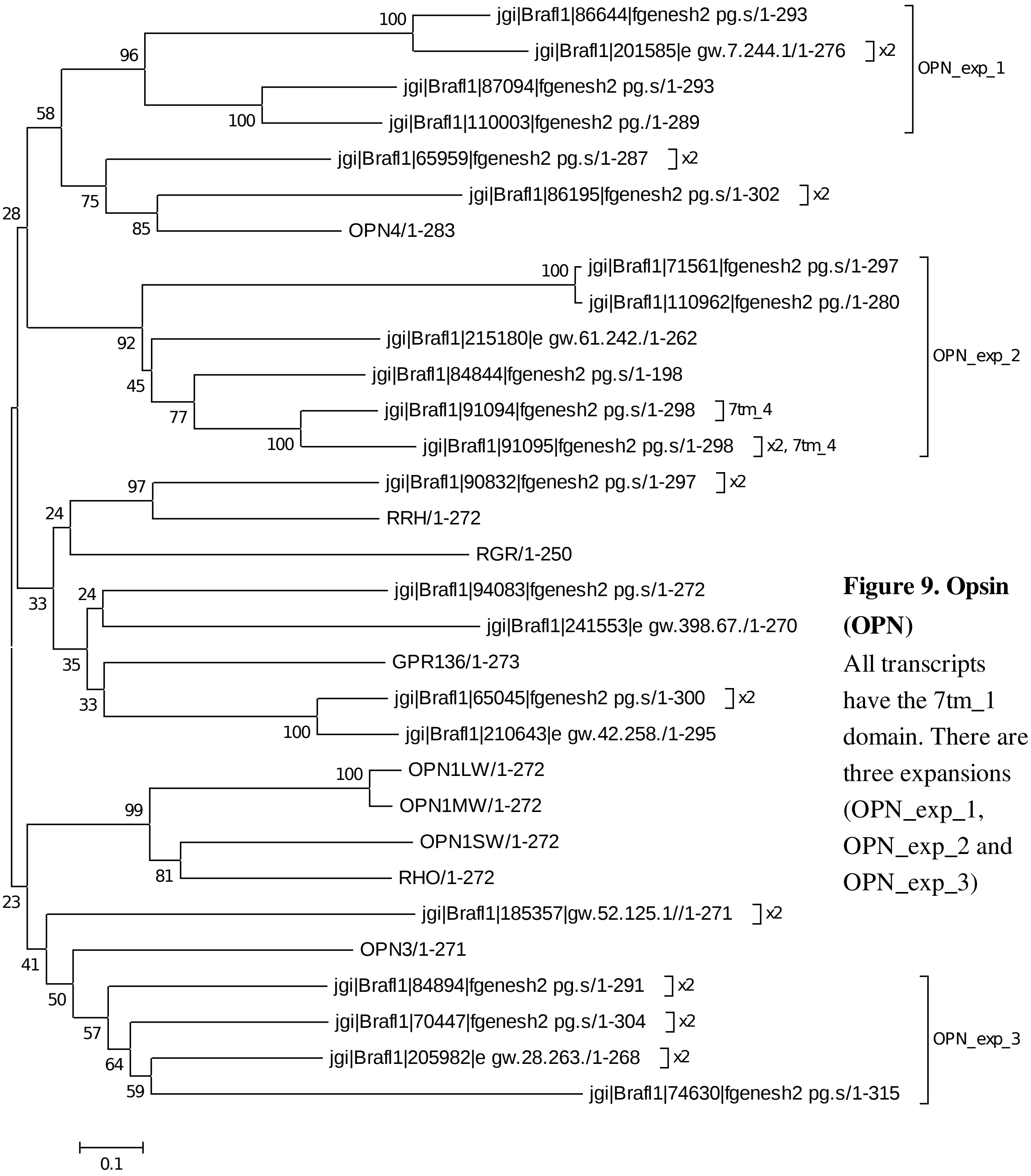

Figure 9. Opsin (OPN) All transcripts have the 7tm_1 domain. There are three expansions (OPN_exp_1, OPN_exp_2 and OPN_exp_3)

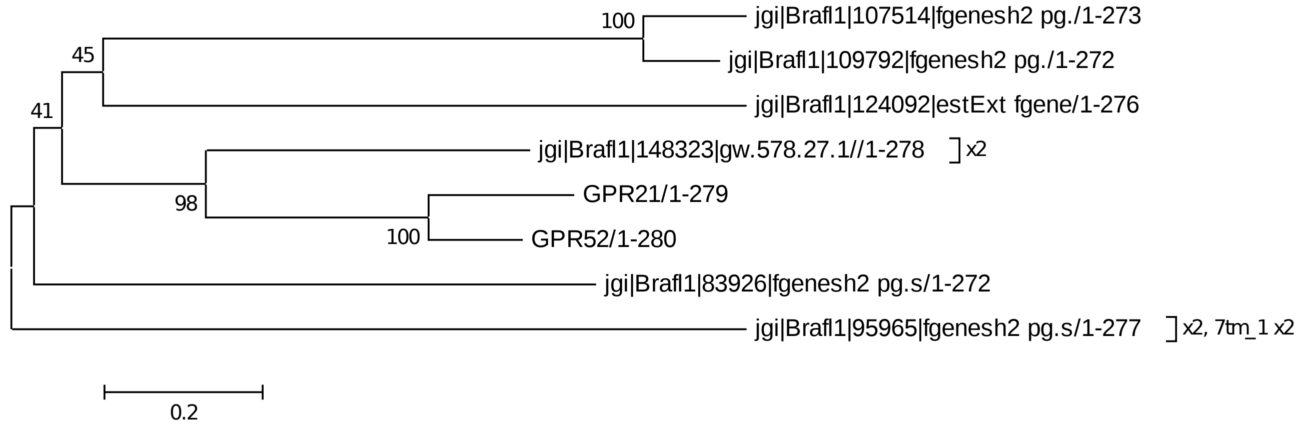

Figure 10. GPR21-GPR52 (OPN subgroup)

All transcripts have the 7tm_1 domian.

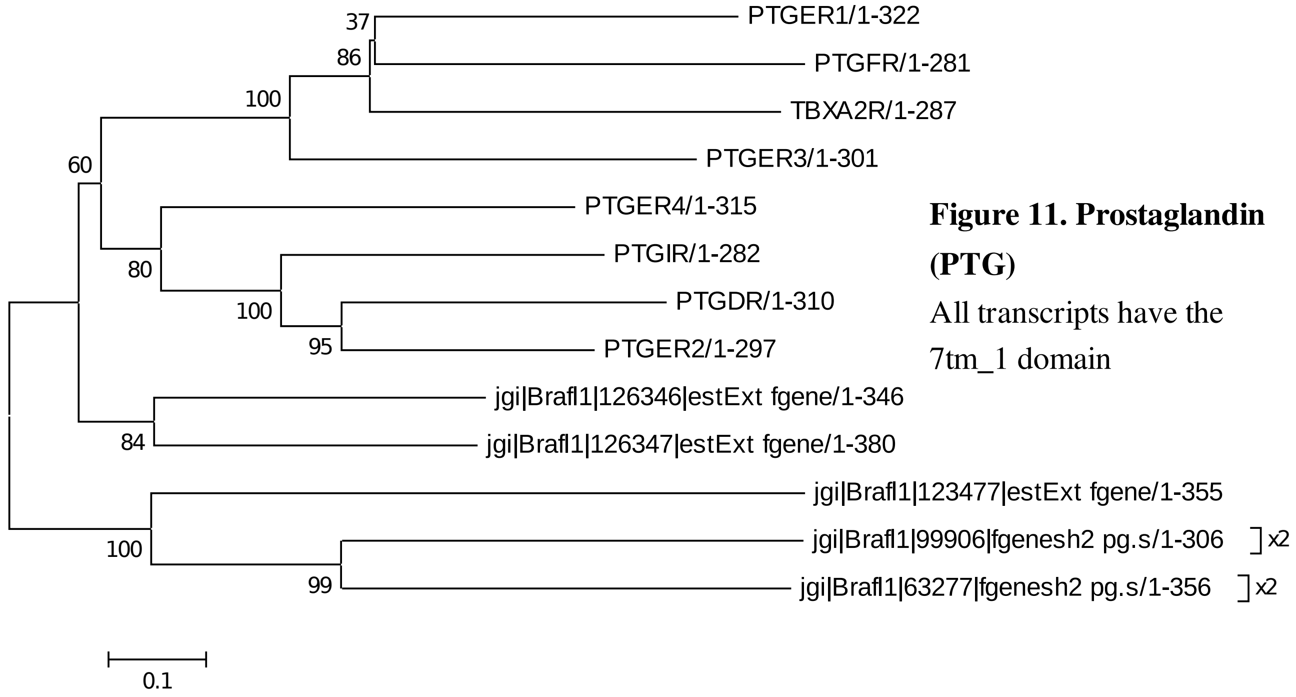

Figure 11. Prostaglandin (PTG)

All transcripts have the 7tm_1 domain

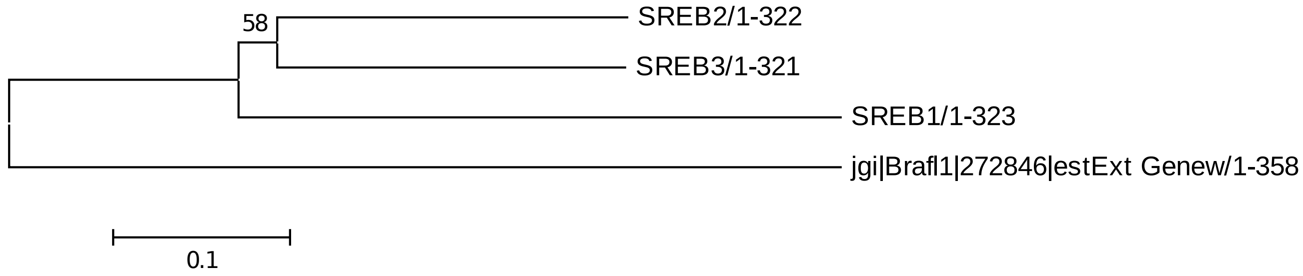

Figure 12. Super conserved receptor expressed in brain (SREB) (PTG subgroup)

All transcripts have the 7tm_1 domain.

Rhodopsin beta

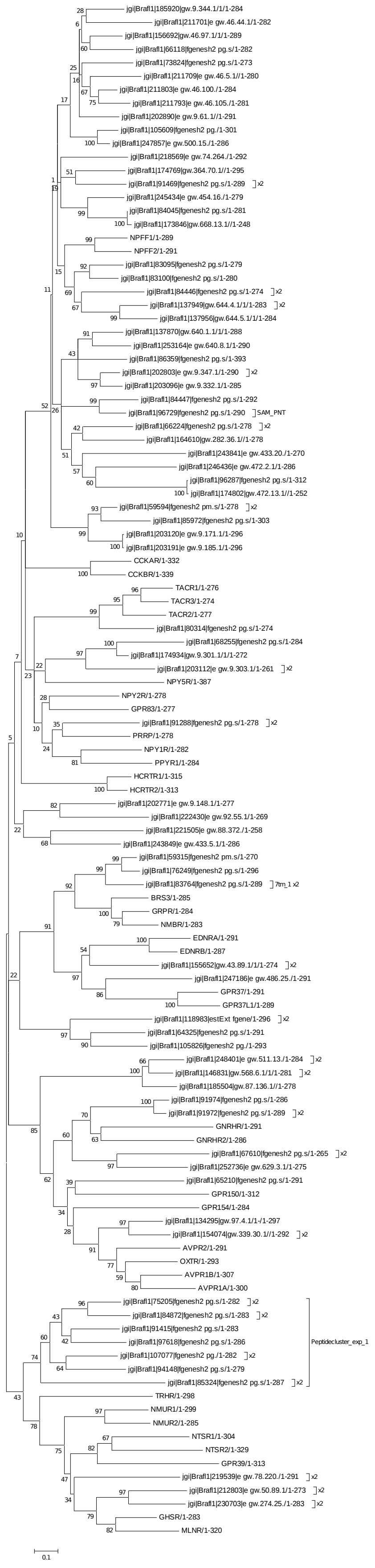

Figure 13. All transcripts have the 7tm_1 domain.

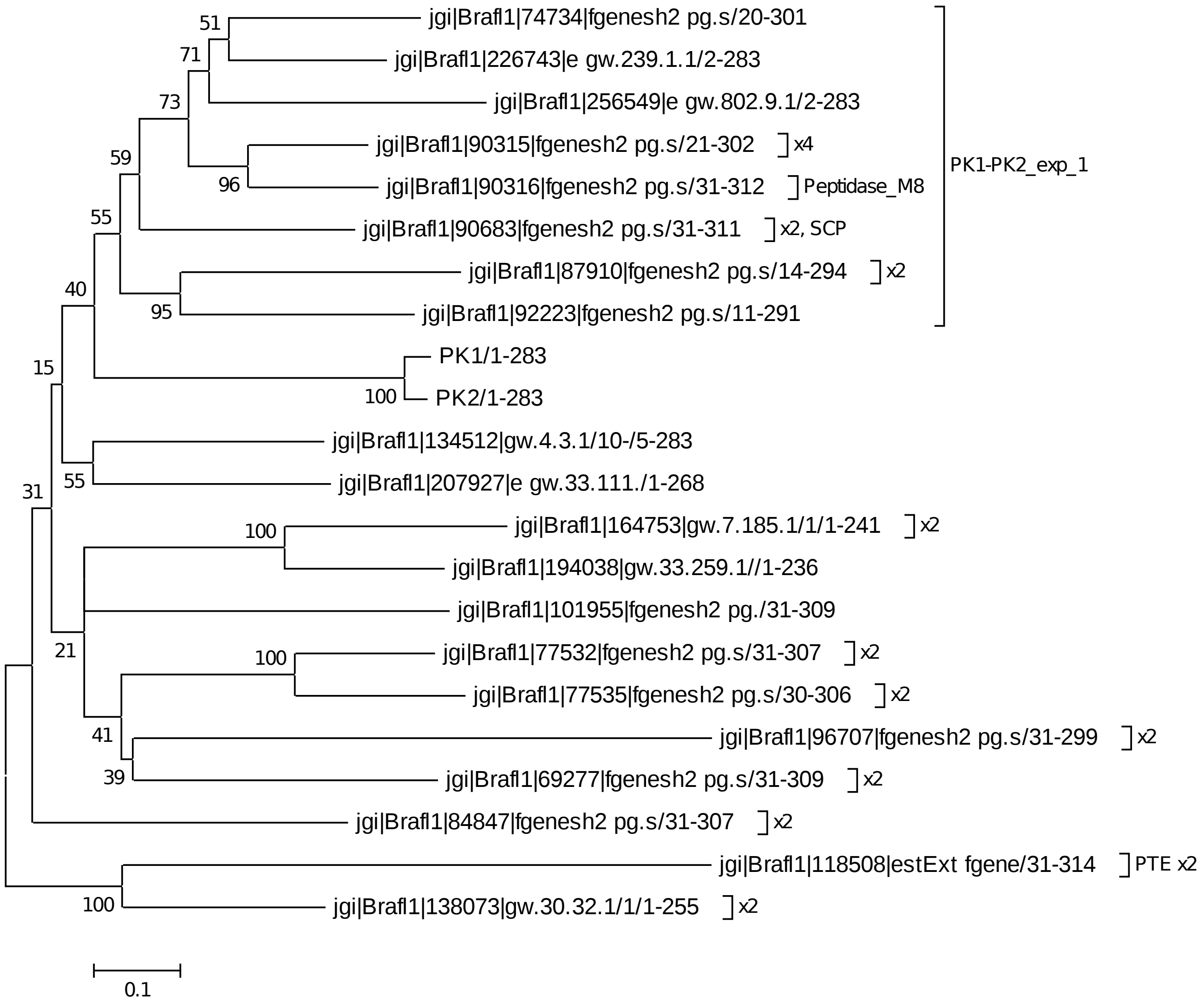

Figure 14. Prokineticin (PK1-PK2) (beta subgroup)

All transcripts have the 7tm_1 domain and there is one expansions (PK1-PK2_exp_1).

Rhodopsin gamma

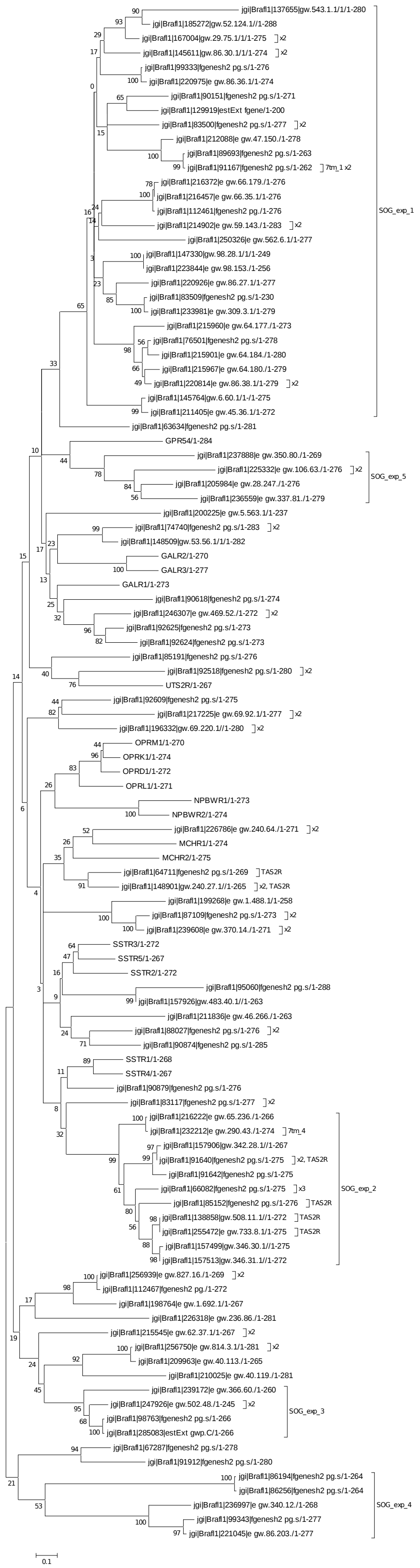

Figure 15. Somatostatin, opioid and galanin (SOG) All transcripts have 7tm_1 domain and there is five expansions (SOG_exp_1, SOG_exp_2, SOG_exp_3, SOG_exp_4 and SOG_exp_5).

Rhodopsin delta

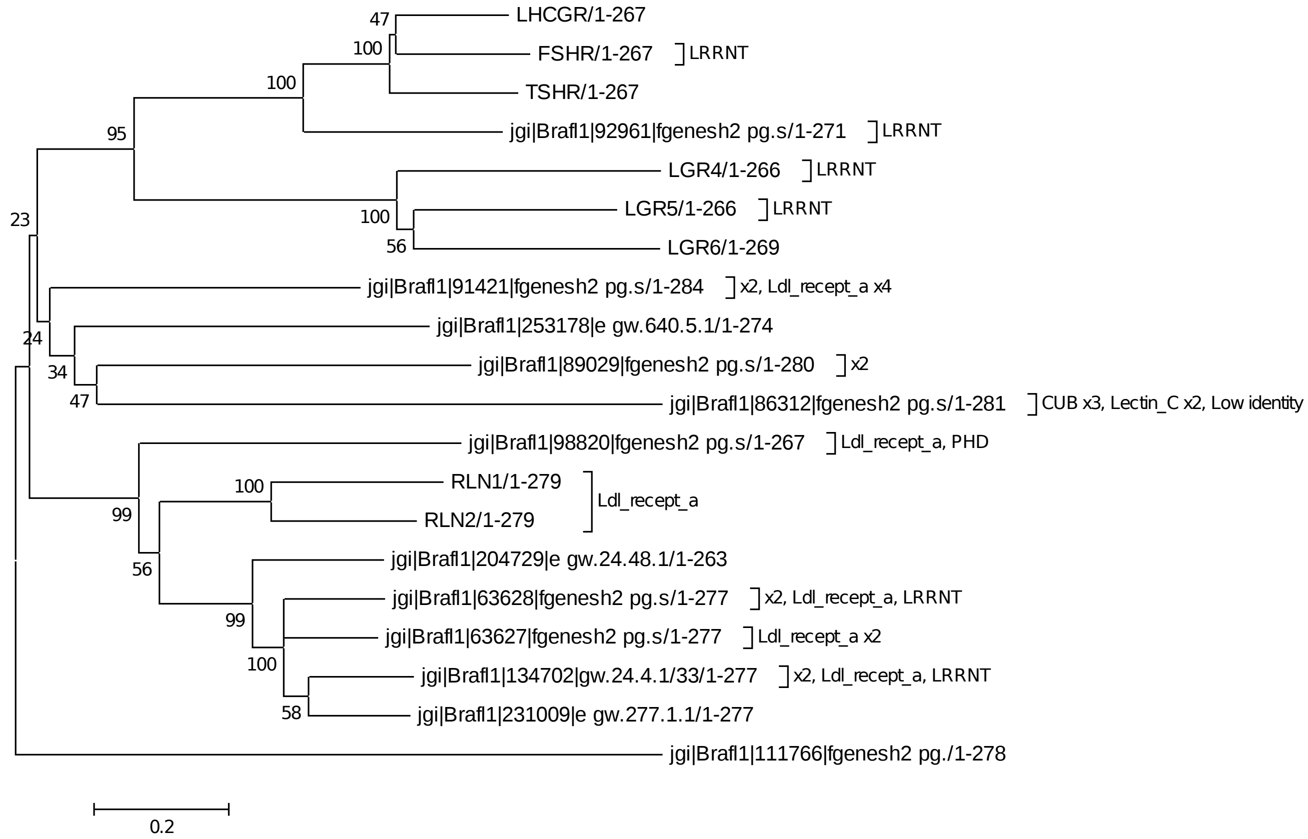

Figure 16. Glycoprotein and leucine-rich-repeat-containing (GP-LGR)

All transcripts have the 7tm_1 domain in addition to what is annotated in the tree.
