# Supplementary material for: The amphioxus (Branchiostoma floridae) genome contains a highly diversified set of G protein-coupled receptors
Source: BMC Evol Biol. 2008 Jan 16;8:9. doi: 10.1186/1471-2148-8-9 (PMC2246102; doi:10.1186/1471-2148-8-9)
Supplement: Additional file 2 — Multiple alignments of the Somatomedin B and the Kringle domains. Two multiple alignments of the Somatomedin B and the Kringle domains found in an expansion of the amphioxus Adhesion family. [file 1471-2148-8-9-S2.pdf]

Additional file 2 – Multiple alignments

Domain searches were made using the HMMER package and the local Pfam models. Interesting domains were cut out in Jalview and aligned, to the appropriate domain sequence. This was done with ClustalW and standard parameters. Sequences for the domains were procured using hmmemit from the HMMER package.

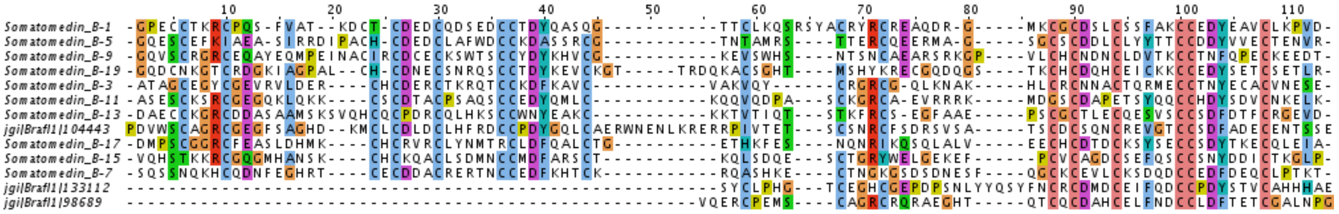

Figure 1. Somatomedin_B: Multiple alignment between three cut amphioxus transcripts and ten double Somatomedin_B domains.

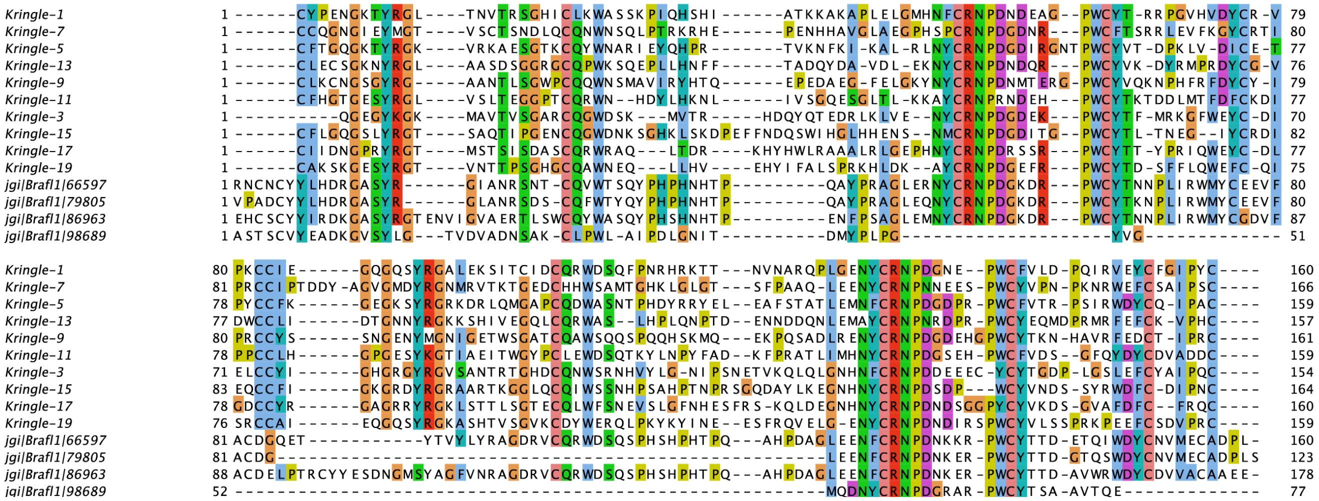

Figure 2. Kringle: Multiple alignment of four amphioxus transcripts and ten double Kringle domains. All but the last have two Kringle domains, which is the reason why the last alignment is gapped in the middle.
