# Supplementary material for: The amphioxus (Branchiostoma floridae) genome contains a highly diversified set of G protein-coupled receptors
Source: BMC Evol Biol. 2008 Jan 16;8:9. doi: 10.1186/1471-2148-8-9 (PMC2246102; doi:10.1186/1471-2148-8-9)
Supplement: Additional file 4 — Amphioxus neuropeptide, gut peptide and food intake receptors orthologs. Compilation of the orthologs to known vertebrate neuropeptide, gut peptide and food intake receptors. [file 1471-2148-8-9-S4.pdf]

Additional file 4

We investigated whether GPCRs from different categories such as neuropeptide-, gut peptide- and food intake receptors, were found in amphioxus. For the first two categories, we also scanned for their respective peptide ligands. The first three columns contain information about the receptors. The first column holds the names of groups of receptors, the second tells the type of receptor and the third is a comment on if they are present in amphioxus. The remaining columns are associated with the peptide ligand with name and e-values from the best hit for searches with BLAST and HMM-models, respectively (a cut off at 0.01).

Table 1. Neuro peptide receptors.

Receptor	Family/Group	comment	Ligand	peptide BLAST	peptide HMM
BDKRB1-2	chem	whole family missing	Bradykinin		
CCKAR, CCKBR	peptide	no close Branchiostoma transcript	CCK/gastrin		
CALCR	secretin	one probable ortholog	Calcitonin gene related peptide	2.00E-005	7.50E-006
CRHR	secretin	clusters togheter with five Branchiostoma transcripts	Corticotropin releasing factor	2.00E-003	1.90E-005
MCH1	~SOG	one or three possible orthologs	Melanin concentrating hormone		
MC4	MEC	no close Branchiostoma transcript	Melanocortin	5.00E-003	2.80E-003
NPY1R, NPY2R, NPY5R	peptide	possible orthologes	Neuropeptide Y	1.00E-006	3.00E-005
OPRD1, OPRK1, OPRL1, OPRM1	SOG	no close Branchiostoma transcript	Opioid peptides	6.00E-004	7.30E-004
HCRTR1-2	peptide	no close Branchiostoma transcript	Orexins	3.00E-009	5.90E-005
SSTR1-5	SOG	possible orthologes	Somatostatin	2.00E-004	1.80E-003
TACR1-3	peptide	one probable ortholog	Tachykinins	6.00E-004	7.10E-003
VIPR, PTHR	secretin	no close Branchiostoma transcript	VIP, PACAP		
AVPR1A, AVPR1B, AVPR2, OXTR	peptide	two possible orthologes	Vasopressin/oxytocin	1.00E-023	1.10E-012

Table 2. Gut peptide receptors. This table also contains a column regarding the location of the receptor in human.

Receptor	Target	Family/Group	comment	Ligand	peptide BLAST	peptide HMM
CCKAR and CCKBR	Pancreas, Gallbladder	peptide	no close Branchiostoma transcript	Cholecystokinin		
GIPR	Pancreas	secretin	no close Branchiostoma transcript	Gastric-inhibitory peptide		
CCKBR	Parietal cells in body of stomach	peptide	no close Branchiostoma transcript	Gastrin		
GRPR	G cells in antrum of stomach	peptide	Clusters together with BRS3 and NMBR which in turn goes together with three Branchiostoma transcripts	Gastrin-releasing peptide		
GLP1R and GLP2R	Liver	secretin	no close Branchiostoma transcript	Glucagon	4.00E-002	2.10E-003
Guanlyl cyclase receptor - not an GPCR	Small and large intestine			Guanylin	6.60E-001	9.10E-003
MLNR	Esophageal sphincter, Stomach, Duodenum	peptide	cluster together with GHSR and two Branchiostoma transcripts	Motilin	1.70E-001	8.20E-004
NTSR	Intestinal smooth muscle	peptide	also in the same cluster as MLNR, but more basal	Neurotensin		6.30E-003
NPY1R, NPY2R and NPY5R	Stomach, Pancreas	peptide	possible orthologes	Peptide YY	8.00E-007	1.40E-005
SCTR	Pancreas, Stomach	secretin	no close Branchiostoma transcript	Secretin	3.90E-002	4.20E-004
SSTR 1-5	Stomach, Intestine, Pancreas, Liver	SOG	possible orthologes	Somatostatin	2.00E-004	1.80E-003
TAC1-3	Enteric neurons	peptide	one probable ortholog	Substance P	6.00E-004	7.10E-003
VIPR1 and VIPR2	Small intestine, Pancreas	secretin	no close Branchiostoma transcript	Vasoactive instestinal peptide		

Table 3. Food intake regulating receptors

Name	GPCR class	comment	Ligand
MC4R	Rhodopsin, alpha group	No obvious ortholog	α-, β-MSH, Agrp
MC3R	Rhodopsin, alpha group	No obvious ortholog	α-, β-MSH, Agrp
CNR1	Rhodopsin, alpha group	No obvious ortholog	Anandamide/cannabinoids
HTR1B	Rhodopsin, alpha group	In a cluster with two BF transcripts	Serotonin
HTR2C	Rhodopsin, alpha group	No obvious ortholog	Serotonin
HTR6	Rhodopsin, alpha group	In a cluster with three BF transcripts	Serotonin
ADRA1B	Rhodopsin, alpha group	In a cluster with one BF transcript	Adrenaline
ADRA2B	Rhodopsin, alpha group	In a cluster with one BF transcript	Adrenaline
ADRB1	Rhodopsin, alpha group	In a cluster with one BF transcript	Noradrenaline
ADRB2	Rhodopsin, alpha group	In a cluster with one BF transcript	Adrenaline
ADRB3	Rhodopsin, alpha group	In a cluster with one BF transcript	Adrenaline
CHRM3	Rhodopsin, alpha group	In a cluster with one BF transcript	Acetylcholine
DRD2	Rhodopsin, alpha group	In a cluster with two BF transcripts	Dopamine
DRD3	Rhodopsin, alpha group	In a cluster with two BF transcripts	Dopamine
H3	Rhodopsin, alpha group	Missing in dataset	Histamine
NPY Y1R	Rhodopsin, beta group	In a cluster with one BF transcript	NPY
NPY Y2R	Rhodopsin, beta group	In a cluster with one BF transcript	NPY, $PYY_{3\text{-}36}$
NPY Y5R	Rhodopsin, beta group	In a cluster with three BF transcripts	NPY
PRRPR (GPR10)	Rhodopsin, beta group	In a cluster with one BF transcript	PRP
GHSR	Rhodopsin, beta group	In a cluster with two BF transcripts	Ghrelin
ORXR1 (HCRTR1)	Rhodopsin, beta group	No obvious ortholog	Orexin A/B
ORXR2 (HCRTR1)	Rhodopsin, beta group	No obvious ortholog	Orexin A/B
NPFF1R	Rhodopsin, beta group	In a cluster with five BF transcripts	Neuropeptide FF
NPFF2R	Rhodopsin, beta group	In a cluster with five BF transcripts	Neuropeptide FF
NMU1R	Rhodopsin, beta group	Basal to a cluster holding three BF transcripts	Neuromedin U
NMU2R	Rhodopsin, beta group	Basal to a cluster holding three BF transcripts	Neuromedin U
CCK1R (CCKRA)	Rhodopsin, beta group	No obvious ortholog	CCK
CCK2R (CCKRA)	Rhodopsin, beta group	No obvious ortholog	CCK, Gastrin
BRS3	Rhodopsin, beta group	In a cluster with three BF transcripts	-
MCH1R (GPR24)	Rhodopsin, gamma group	In a cluster with one BF transcript	MCH
GPR7	Rhodopsin, gamma group	No obvious ortholog	Neuropeptide W/B
GALR1	Rhodopsin, gamma group	In a cluster with four BF transcripts	Galanin
GALR2	Rhodopsin, gamma group	In a cluster with two BF transcripts	Galanin
GALR3	Rhodopsin, gamma group	In a cluster with two BF transcripts	Galanin/GALP
OPRM1	Rhodopsin, gamma group	No obvious ortholog	β-endorphin
OPRK1	Rhodopsin, gamma group	No obvious ortholog	Dynorphin
FFA1R, (GPR40)	Rhodopsin, gamma group		Medium and long chain carboxylic acids
FFA3R, (GPR41)	Rhodopsin, gamma group		Short chain fatty acids
FSHR	Rhodopsin, delta group	In a cluster with one BF transcript	FSH
TRHR	Rhodopsin, delta group	Whole cluster missing	TRH
GCGR	Secretin	No obvious ortholog	Glucagon
GLP1R	Secretin	No obvious ortholog	GLP
CRH1R (CRF1R)	Secretin	In a cluster with five BF transcripts	CRH, Urocortin
CRH2R (CRF2R)	Secretin	In a cluster with five BF transcripts	Urocortin
